# Blind Source Parameters for Performance Evaluation of Despeckling Filters

**DOI:** 10.1155/2016/3636017

**Published:** 2016-05-19

**Authors:** Nagashettappa Biradar, M. L. Dewal, ManojKumar Rohit, Sanjaykumar Gowre, Yogesh Gundge

**Affiliations:** ^1^Bheemanna Khandre Institute of Technology, Bhalki 58532, India; ^2^Indian Institute of Technology Roorkee, Roorkee 247667, India; ^3^Postgraduate Institute of Medical Education and Research, Chandigarh 160 012, India

## Abstract

The speckle noise is inherent to transthoracic echocardiographic images. A standard noise-free reference echocardiographic image does not exist. The evaluation of filters based on the traditional parameters such as peak signal-to-noise ratio, mean square error, and structural similarity index may not reflect the true filter performance on echocardiographic images. Therefore, the performance of despeckling can be evaluated using blind assessment metrics like the speckle suppression index, speckle suppression and mean preservation index (SMPI), and beta metric. The need for noise-free reference image is overcome using these three parameters. This paper presents a comprehensive analysis and evaluation of eleven types of despeckling filters for echocardiographic images in terms of blind and traditional performance parameters along with clinical validation. The noise is effectively suppressed using the logarithmic neighborhood shrinkage (NeighShrink) embedded with Stein's unbiased risk estimation (SURE). The SMPI is three times more effective compared to the wavelet based generalized likelihood estimation approach. The quantitative evaluation and clinical validation reveal that the filters such as the nonlocal mean, posterior sampling based Bayesian estimation, hybrid median, and probabilistic patch based filters are acceptable whereas median, anisotropic diffusion, fuzzy, and Ripplet nonlinear approximation filters have limited applications for echocardiographic images.

## 1. Introduction

Echocardiography is commonly used in the diagnosis of valvular regurgitation and stenosis. It is a noninvasive, safe, and a less expensive technique; but the low contrast, shadowing, and the speckle noise present make it hard for a clinician to read. The noise masks the finer clinical detail present in the image and thereby reduces the human visual ability for detecting the abnormalities. It reduces the potentiality of images in providing crucial and vital information [1]. The interpretation and clinical conclusions largely depend on the image quality and the experience of the cardiologist [1, 2]. Hence, it is necessary to suppress speckle noise without altering the fine details from the transthoracic echocardiographic (TTE) images.

Filtering techniques based on the concepts such as wavelet [1, 3–6], anisotropic diffusion (AD) [1, 4, 5], a priori statistical information [1, 4, 7, 8], multiresolution [9–13], nonlocal means (NLM) [4, 14, 15], total variation (TV) [16–18], bilateral [19, 20], median [5–7], Wiener [5, 13], geometric [1, 5], local statistics [5], and fuzzy [21] filters have been advocated for noise reduction in ultrasound images. Various filtering techniques and performance parameters used by researchers are tabulated in Table 1. Most of the researchers have employed standard full-reference based metrics such as peak signal-to-noise ratio (PSNR), mean square error (MSE), structural similarity (SSIM) index, contrast-to-noise ratio (CNR), and root mean square error (RMSE), tabulated in Table 1, for evaluating the filter performance.

These full-reference parameters evaluate the filter performance considering the output processed and the standard noise-free image. But, unfortunately, the noise-free reference image does not exist for echocardiographic images. Hence, these traditional parameters do not reflect the true performance in case of the echocardiographic images. The possible solution to this problem would be to use parameters such as speckle suppression index (SSI) [7, 8], speckle suppression and mean preservation index (SMPI) [7, 8], and beta metric (*β*) [6, 21]. These are blind source based parameters which do not need a standard noise-free reference image for estimating filter performance. The speckle noise present in echocardiographic images is multiplicative in nature. This noise masks the finer details necessary for abnormality diagnosis. Many filters result in image smoothing thereby suppressing the finer details. Hence, it would be necessary to know those of the filters which can suppress the noise while preserving the finer details.

Mateo and Fernández-Caballero [6] and Biradar et al. [21] employed beta metrics (*β*) to evaluate edge preservation. Iqbal et al. [8] used SSI and SMPI to measure amount of speckle suppression and edge preservation index (EPI) for estimating edge preservation in synthetic aperture radar (SAR) images. This paper evaluates the performance of eleven types of filters based on SSI, SMPI, *β*, figure of merit (FoM), and image quality index (IQI) and eleven other parameters along with the visual quality assessment and clinical validation. A list of filters types and their constituent approaches is tabulated in Table 2 for quick reference.

## 2. Materials and Methods

The speckle is a multiplicative and locally correlated noise. The speckle noise stems from point scatterers in a homogenous tissue, which cannot be resolved by the ultrasound machine. The point scatterers, which are much smaller than the ultrasound wavelength, scatter the wave. Two or more waves travelling towards the scanning probe from such scatterers may interfere with each other, constructively or destructively, creating bright and dark spots, which are commonly known as speckles. For interference, the backscattered signal from the scatterers should overlap in time and space. This happens when the distance between them is within the point spread function (PSF) support. It is important to note that the typical size of the speckle is similar to the PSF support. As the PSF changes with depth, the statistics of speckle noise are space (depth)-variant. To find a clean approximation of the spatial reflectance distribution, the point spread function (PSF) of the imaging system is being estimated. The noisy degraded images create a problem for clinicians to discriminate fine details of the image during diagnosis. The speckle fluctuations would be proportional in magnitude to signal strength and the resultant image will have significant noise even in the bright sections of the image. The noise is modeled as

(1) $$\begin{array}{c} f\left(\;x,y\;\right)=g\left(\;x,y\;\right)n\left(\;x,y\;\right), \end{array}$$

where *f*(*x*, *y*) is the original noisy image, *g*(*x*, *y*) is the noise-free image, *n*(*x*, *y*) is the multiplicative noise, and (*x*, *y*) is the pixel locations in each of the images [3, 6, 9]. The variables *x* and *y* are the spatial pixel locations. The multiplicative noise is converted into approximate additive noise by applying logarithmic transformation on both sides of (1):

(2) log⁡fx,ylog⁡gx,ynx,y=log⁡gx,y+log⁡nx,y.

Equation (2) can be rewritten as *f*_*xy*_ = *g*_*xy*_ + *n*_*xy*_, where *f*_*xy*_ = log⁡[*f*(*x*, *y*)], *g*_*xy*_ = log⁡[*g*(*x*, *y*)], and *n*_*xy*_ = log⁡[*n*(*x*, *y*)].

### 2.1. Despeckling Filters

Loizou et al. [5, 22] compared applications of 10 despeckling filters for suppression of speckle noise in US images of carotid artery. This paper studies the applications of despeckling filters (DsFs) considered by Loizou et al. [5, 22] for TTE images. The despeckling filter based on the mean and the variance of the neighborhood is abbreviated as DsFlsmv where DsF stands for despeckling filter and ls, m, and v represent local statistics, mean, and variance, respectively.

The output of first-order local statistics filter is given by

(3) $$\begin{array}{c} f_{\text{denoised}}={\overset{-}{g}}_{x,y}+W_{x,y}\left(\;g_{x,y}-{\overset{-}{g}}_{x,y}\;\right), \end{array}$$

where ${\overset{-}{g}}_{x,y}$ is the local mean, *f*_denoised_ give the estimated denoised pixel values in the window, and *W*_*x*,*y*_ is the weighing factor [5, 22], where *W* ∈ [0,…, 1]. The weighing factor used in DsFlsmv filter is defined as

(4) $$\begin{array}{c} W_{x,y}=\frac{\sigma^{2}}{\sigma^{2}+{{\overset{-}{g}}_{x,y}}^{2}\sigma_{n}^{2}}, \end{array}$$

where *σ* ^2^ is the variance of the moving window, *σ*_*n*_^2^ is the ratio between variance and mean computed on the whole image, and $\sigma_{n}^{2}=\sum_{x=1}^{p}\sigma_{p}^{2}/{\overset{-}{g}}_{p}$ with *σ*_*p*_^2^ and ${\overset{-}{g}}_{p}$ as the variance and the mean of noise selected in the window [5, 22]. The homogenous mask area filtering technique is abbreviated as DsFlsminsc filter (DsF-local statistics minimum speckle index). It works on the most homogeneous 5 × 5 neighborhood around each pixel using a subset of 3 × 3 window. The Wiener filter is based on the mean square error and it is abbreviated as DsFwiener [5, 22]. The weight factor of DsFwiener filter is given by

(5) $$\begin{array}{c} W_{x,y}=\frac{\left(\sigma^{2}-\sigma_{n}^{2}\right)}{\sigma^{2}}. \end{array}$$

The filter based on the estimation of maximum homogeneity over a pixel neighborhood is called the DsFhomog filter. This filter estimates the homogeneous neighborhood around every pixel by taking into account pixels belonging to the processed neighborhood. The hybrid median is abbreviated as DsFhmedian (despeckling filter-hybrid median). It is an extension of DsFmedian (despeckling filter-median). The median values are calculated using three different windows, namely, normal shape, x-shape, and cross shape [5, 22].

### 2.2. Synthetic Aperture Radar (SAR) Filters

The standard adaptive SAR filtering techniques such as those of Lee, Kuan et al., and Frost et al. are analyzed in this paper [1, 7, 8, 23]. The formulations of Lee and Kuan et al.'s filter are similar but with different weighting function. In Frost et al.'s filter, the noise-free image is being computed by convolving the original noisy image with a mask.

### 2.3. Fast Bilateral Filter

The nonlinear characteristics of range filter in the bilateral filter increase the computational load. An improvement in the performance can be achieved using the raised cosine kernels. The generalized form of the filter based on the raised cosine kernel is given by

(6) $$\begin{array}{c} \hat{f}\left(\;x\;\right)=\frac{\sum_{\left|n\right|\leq M}d_{n}\left(x\right){\overset{-}{g}}_{n}\left(x\right)}{\sum_{\left|n\right|\leq M}d_{n}\left(x\right){\overset{-}{h}}_{n}\left(x\right)} \end{array}$$

with *d*_*n*_(*x*) = *c*_*n*_exp⁡(−*jnγf*(*x*)), where *c*_*n*_ is the coefficient of trigonometric function, *M* is kernel degree, and *h*_*n*_(*x*) = exp⁡(*jnγf*(*x*)) and *g*_*n*_(*x*) = *f*(*x*)*h*_*n*_(*x*) represent the auxiliary images and range kernel.

### 2.4. Fuzzy Filter

The fuzzy filters based on the triangular function with median (TMED) center, asymmetrical triangular function with median (ATMED), triangular moving average (TMAV) center, and asymmetrical triangular moving average (ATMAV) are analyzed in the logarithmic domain in this paper. The output of the fuzzy filter is given by

(7) fdenoisedx,y=∑r,s∈AFfx+y,y+s·fx+r,y+s∑r,s∈AFfx+r,y+s,

where *F*[*f*(*x*, *y*)] are the window functions based on TMED, ATMED, TMAV, or ATMAV and *A* is the area [21, 24]. All the four logarithmic fuzzy filters are combined with Wiener filter and are known as hybrid fuzzy filters. The performances of hybrid fuzzy filters are further improved by embedding modified geometric filter. The filters are abbreviated as the GWF filters where G stands for geometric, W stands for Wiener, and F stands for fuzzy filters [21, 24].

### 2.5. Fourier Filtering

The images are transformed from the spatial domain to the frequency domain and vice versa in Fourier filters. Fourier Butterworth filter (FBF) reduces the noise with the edges preserved. The homomorphic Fourier ideal filter (HFIF) and the homomorphic Fourier Butterworth filter (HFBF) are implemented by projecting the input image into the logarithmic space, applying the fast Fourier transformations, filtering the image using ideal or Butterworth filter, followed by inverse FFT transformations, and projecting the image back into the nonlogarithmic space [6].

### 2.6. Wavelet Shrinkage Techniques

The wavelet shrinkage techniques can be used in despeckling applications by operating in the logarithmic domain. The input is projected into logarithmic space, subjected to denoising, and projected back to the nonlogarithmic space. This procedure can be mathematically represented as *f*_denoised_(*x*, *y*) = exp⁡[*D*_*x*_(log⁡(*f*(*x*, *y*) + 1))] − 1, where *D*_*x*_ are the wavelet shrinkage techniques [25–29]. During the implementation of wavelet shrinkage in the logarithmic domain, it is assumed that the noise variance is known, so that the focal point of discussion would be on the evaluation of the filter itself. The Bayes thresholding approach is a data driven subband adaptive wavelet shrinkage technique [25]. The concept of the multiscale product thresholding (MPT) is based on the idea that multiplications of the discrete wavelet transform (DWT) at adjacent scales enhance the edges and effectively suppress the noise [27]. In logarithmic probability shrinkage (ProbShrink) filtering method, the wavelet coefficients are multiplied with the estimated probability of the signal [21, 30]. In SURELET [29], the pointwise thresholding is performed based on the minimization of SURE and LET. The interscale orthonormal wavelet thresholding (IOWT) is a parameterized denoising technique which alleviates the necessity of a statistical design model [26]. The limitations of block thresholding are overcome by incorporating SURE with NeighShrink resulting in NeighShrinkSURE (NSS) [28]. The details subbands are extracted and subband thresholding is reinforced with calculation of the optimal threshold and neighborhood size followed by thresholding using NeighShrink.

### 2.7. Multiresolution Techniques

Some of the multiresolution based filtering techniques such as M-band ridgelet (MBR) [9, 21], Ripplet nonlinear approximation (RNLA) [11, 12], generalized likelihood estimation method (GLM) [1, 4, 13], and posterior sampling based Bayesian estimation (PSBE) [10] are considered in this paper for performance evaluation. The combination of M-band wavelet and ridgelet is known as M-band ridgelet (MBR). The texture is preserved using MBR filter [9, 21]. In the GLM based filter, an initial classification of the coefficients is carried out based on the correlation among the prominent features across various resolution scales noniteratively. The initial classification is employed for the estimation of statistical distribution of the features of interest [1, 4, 13]. In the logarithmic PSBE, noise-free image details are estimated using Bayesian least square error calculations using conditional posterior sampling and then the average square error is minimized [10]. The generalization of curvelets known as Ripplet transforms is being achieved by incorporating support and degree parameters [11, 12]. The Ripplet transformation in discrete domain is defined as

(8) $$\begin{array}{c} R_{j,\vec{k},l}=\overset{M-1}{\underset{n_{1}=0}{\sum }}\;\overset{N-1}{\underset{n_{2}=0}{\sum }}f\left(\;n_{1},n_{2}\;\right)\bar{\rho_{j,\vec{k},l}\left(n_{1},n_{2}\right)}, \end{array}$$

where the Ripplet coefficients are represented by $R_{j,\vec{k},l}$ [11, 12].

### 2.8. Total Variation (TV) Based Filters

The TV based denoising is based on the concept that the integral of absolute gradient of the noisy images would be high resulting in high total variation [18]. Let *f* be a noisy image; the denoised image *f*_denoised_ can be obtained by minimizing the quadratic term with the TV regularization given by

(9) $$\begin{array}{c} f_{\text{denoised}}=\underset{u}{\arg\;\min}\int_{\Omega }{\left(\;f-u\;\right)}^{2}dx+\lambda \int_{\Omega }\left|\;\nabla f\;\right|dx, \end{array}$$

where the quadratic data term ∫_*Ω*_(*f* − *u*)^2^ *dx* fits *f* in *u* according to the least square fit, ∫_*Ω*_|∇*f*|*dx* is the regularization term (denoising function), and *λ* is the weighting parameter which is the measure of smoothness. The fidelity term controls the amount of denoising by measuring local variance in the image in adaptive fidelity based TV (AFTV) [16]. Anisotropic TV (ATV) is based on split Bregman algorithm. The Bregman iterations are employed during the denoising process [17].

### 2.9. Anisotropic Diffusion (AD) Based Filters

The AD is a nonlinear partial differential equation (PDE) based filtering technique that promotes diffusion in the homogeneous regions while holding back at edges [1, 31]. In speckle reducing anisotropic diffusion (SRAD) filter, the diffusion function is controlled by instantaneous coefficient of variation (ICOV) [1, 32]. The diffusion function employed in SRAD is of the form represented in

(10) cqx,y;t,q0t=1+q2x,y,t−q02tq2x,y,t1+q02t−1,

where *q*_0_ stands for the “speckle scale function.” The coherence enhancing diffusion (CED) employs structure tensor matrix for analyzing the local distribution of gradient vector [1, 33]. Detail preserving anisotropic diffusion (DPAD) method estimates equivalence between threshold controlling the level of diffusion and variation in noise coefficient by incorporating modifications to the SRAD filter [4, 34].

### 2.10. Geometric Filter

The geometric filter works with an assumption that the images are made up of the valleys and the narrow walls. An intensity of pixel located at the center of a 3 × 3 window is compared with eight neighbors. Depending on the intensity values of the neighborhood pixels, the value is either incremented or decremented so that the values stand out in comparison with the intensity value in the neighborhood [1, 35].

### 2.11. Nonlocal Mean Filter

The nonlocal means approach of denoising is governed by the patches around each pixel. The redundant information in the image is being used in optimized Bayesian nonlocal means (OBNLM) [4, 14]. The patterns surrounding each pixel are compared instead of the intensity. In NLM based denoising, the Euclidean distance is computed between the patches. The generalization of this distance was advocated using probabilistic patch based (PPB) method [15]. The weight *w*(*s*, *t*) in probabilistic patch based (PPB) filter between patch *s* and patch *t* with *i* number of iterations is defined as

(11) ws,t=exp⁡−∑k2L−1hlog⁡fs,kft,k+ft,kfs,k+1Tf^s,ki−1−f^t,ki−12f^s,ki−1f^t,ki−1,

where trade-off between noise suppression and fidelity estimation is achieved through parameters *h* and *T*; *f*_*t*,*k*_ and *f*_*s*,*k*_ are the *k*th pixel amplitude, where their previous values are ${\hat{f}}_{t,k}^{i-1}$ and ${\hat{f}}_{s,k}^{i-1}$ for patches *t* and *s*, respectively.

## 3. Evaluation of Despeckling Filters

The despeckling filters are analyzed and evaluated for the clinical TTE images. These images are noisy; no noise-free reference image is available. Therefore, the evaluation of filters is carried out using the original speckled input. The analysis of performance is based on the blind assessment and the full-reference based image quality metrics. The blind assessment parameters such as the SSI [7, 8], SMPI [7, 8], and *β* [6, 21] are used in performance evaluation. These parameters assess the performance based on the original noisy image and the denoised images. The parameters such as the SSIM, IQI, and FoM [1, 4, 21] are used for measuring overall image quality, structure, and edge preservation. The traditional parameters such as PSNR, MSE, SNR, LMSE, RMSE, average difference, and maximum difference (MD) are also being computed using original TTE images. The traditional parameters may not reflect the true performance of the filters on real clinical images; therefore, the evaluation of performance is being validated by the practicing clinicians. All the experiments are implemented using MATLAB R2008a. The source codes provided by the authors of various papers are also being used with proper selection of parameters [5, 11, 13–17, 20, 25–30].

### 3.1. Clinical Echocardiographic Images

The despeckling filters are applied to clinical database consisting of the TTE images of aortic valve and cardiac chambers. The database consists of images acquired in parasternal long axis (PLAX), parasternal short axis (PSAX), apical five-chamber (A5C), apical four-chamber (A4C), and apical two-chamber (A2C) views. The TTE image database was completely anonymized, with patient information provided. A total of 1000 images acquired from 20 patients in 5 views and 2 windows are used to evaluate and analyze the performance of despeckling filters.

### 3.2. Image Quality Metrics

The parameters such as the SNR, PSNR, MSE, correlation coefficient (*ρ*), RMSE, SC, LMSE, MD, Err3, Err4, NAE, NCC, and SSIM are computed using noisy and noise-free images [5, 22]. Some of the performance parameters are defined below:

(12) SSI=VARfdenMeanfdenMeanforgVARforg,SMPI=Q×VARfdenVARforg,

where *Q* = *K* + |Mean(*f*_den_) − Mean(*f*_org_)|, *K* = (max⁡(Mean(*f*_den_)) − min⁡(Mean(*f*_den_)))/Mean(*f*_org_),

(13) β=DΔfden−Δ−forg,Δforg−Δ−forgDΔfden−Δ−fden,Δfden−Δ−fden·DΔforg−Δ−forg,Δforg−Δ−forg,FoMfden,fref=1max⁡Nden,Nref∑j=1Nden11+γdj2,

where *γ* is the scalar multiplier being utilized as penalization factor with typical value 1/9, *N*_den_ and *N*_ref_ are the number of pixels in original and processed images, respectively, *d*_*j*_ is the Euclidean distance, Δ*f*_den_ and Δ*f*_org_ represent the filtered version of original and processed images, and pixel mean intensities in the region Δ*f*_den_ and Δ*f* are represented by $\overset{-}{\Delta }f_{\text{den}}$ and $\overset{-}{\Delta }f_{\text{org}}$, respectively [1, 6–8].

## 4. Results

The parameters being used in the implementation of filters are tabulated in Table 3. The results are being tabulated for each type of filter so that performance can be analyzed for intra- and interfilter type. The values of image quality metrics obtained on application of various filters are tabulated in Tables 4 [Table tab5]–6. The results for wavelet shrinkage and multiresolution techniques are tabulated in Table 4. The experimental results for despeckling, Fourier, and SAR filters are tabulated in Table 5.

*Wavelet Shrinkage Techniques*. The wavelet shrinkage techniques such as the MPT, BayesShrink, OWT, BlockShrink, SURELET, and NSS result in better texture preservation compared to SAR filters. Wavelet soft thresholding is inferior in terms of *β* and SMPI. Maximum speckle noise is suppressed using DB45 and DMEY mother wavelets. The values of SMPI are very high (≤40) suggesting that speckle is not suppressed using wavelet soft thresholding. All the wavelet soft shrinkage techniques result in FoM value in the range of 0.4 to 0.8. *β* computed for soft thresholding is <0.01 reflecting the loss of edge information.

The noise is suppressed effectively using log NSS filter. The log NSS filter and the ProbShrink (PS) filter result in the best values of SSI among the multiscale techniques. The IOWT based denoising results in SSI ≥1.1 which suggests that the speckle noise is not suppressed.

*Multiresolution Techniques*. The multiresolution filtering techniques such as MBR, RNLA, PSBE, and GLM based filters perform well compared to most of the shrinkage techniques. The GLM based filtering approach is effective in the noise suppression and the edge preservation as reflected by IQI (0.7), *β* (0.9), FoM (0.9), SMPI (2.5), and SSI (0.99). The logarithmic PSBE and logarithmic MPT filters result in effective noise suppression and edge preservation, similar to the GLM based filter. It is also observed that the SMPI values for PSBE, GLM, RNLA, and MBR are superior in comparison to ProbShrink. The PSBE and GLM based filters result in *β* to be approximately equal to one.

*Despeckling Filtering Techniques*. The performances of the DsFlsmv, DsFwiener, DsFmedian, and DsFsrad filter are similar in terms of IQI, FoM, SMPI, and SSI. The SMPI of the DsFgf4d filter is double that of the DsFwiener, DsFmedian, and DsFsrad filter, which reflects its inferiority in terms of speckle suppression. The DsFhomog filter has the SMPI which is almost 4 times higher compared to DsFlsmv and DsFsrad filter. The SSI of all the DsFs (except DsFhomog) is less than one and the DsFls filter has the lowest values. The edges are not preserved using the DsFlsminsc and DsFhomo whereas the DsFlsmv, DsFwiener, and DsFwaveltc filters preserve edges as shown by *β* ≥ 0.9.

*Fourier*,* SAR, and Fuzzy Filters*. The performances of the HFIF and the HFBF filters are superior in comparison to the FIF and FBF filters in terms of SSI. These results in terms of SMPI values are superior by a factor of two compared to SAR filters. FoM is greater than one suggesting better denoising performance. Homomorphic Fourier filters result in smaller value of *β* compared to Fourier filters. Fourier filters have *β* ≥ 0.9 which speak of edge preservation. The issue of concern is smaller IQI using Fourier based techniques. It is observed that IQI is less than 0.4 using the FIF and FBF filters. The SAR filters oversmooth the texture present in the TTE images. The values of SSI are greater than one using Lee, Kaun et al., and Frost et al.'s filters indicating that speckle noise is not suppressed. The performance of SAR filters is poor in terms of SMPI. The major problems using SAR filters are high values of SMPI and SSI. The fuzzy filters perform well in terms of IQI, FoM, and SSI with fractionally higher SMPI (≤4) but have poor beta metric. The GW filter results in the least SSI value among all the fuzzy and hybrid fuzzy filters. The hybrid fuzzy filters have smaller SMPI compared to logarithmic fuzzy filters. All fuzzy and hybrid fuzzy filters have FoM greater than 0.8. The modified geometric filter preserves the edges as exhibited by *β* ≥ 0.9.

*Total Variation*,* Diffusion, and Nonlocal Mean Based Filters*. The performance of TV based filters is moderate in terms of IQI and SMPI. The AFTV and ATV filters result in poor IQI (≤0.3) and moderate *β*, FoM, and SMPI. These filters have SSI <1. All the TV filters have FoM greater than 0.8 except for the AFTV filter. Filters such as AFTV and TV have *β* ≤ 0.5. The output images on application of PPB and NLM filters result in SSI ≤1. The DPAD, FBL, NLM, and PPB filters have SMPI far less compared to SAR filters proving their superior speckle suppression capabilities. The FoM using the DPAD and PPB filters is almost equal to one and is far superior compared to the SAR and OBNLM filters. The IQI is comparatively less using the PPB (0.4), NLM (0.6), and DPAD (0.6) filters whereas FBL (0.7) is fractionally higher.

The performances in terms of IQI, *β*, FoM, SMPI, SSI, and MD for 1000 TTE images are tabulated in Table 7. The performances of the filters are also evaluated in terms of traditional parameters and the values are tabulated in Table 8. The results are being represented in the form of average plus/minus standard deviation. The DsFwiener, DsFsard, GLM, PPB, GWF, DPAD, and PSBE stand out among the others in Table 8.

**Figure 1:**
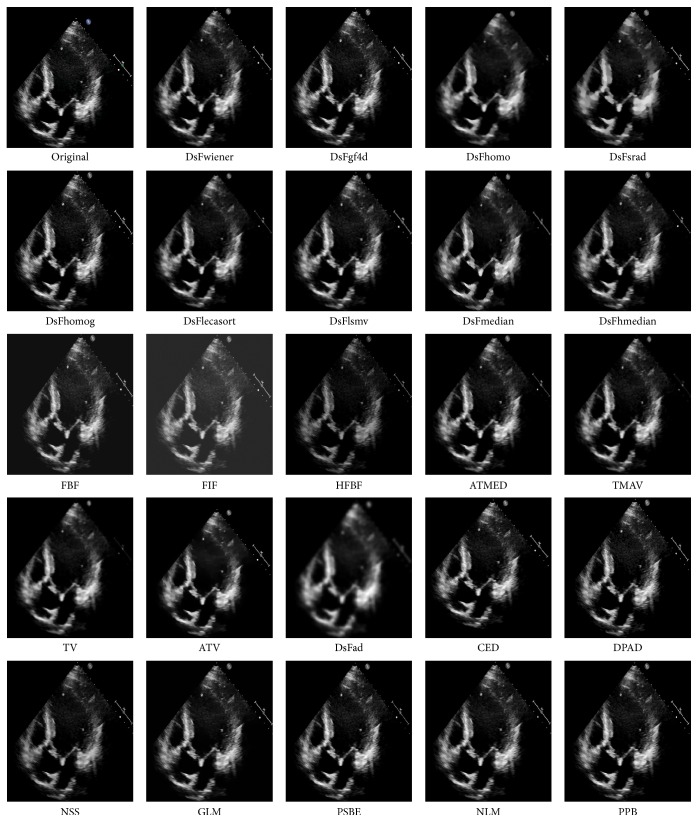
Visual quality of TTE images on application of various filters.

*Visual Quality Assessment*. The visual qualities of denoised A5C TTE image are shown in Figure 1. The blocky effect appears in Lee and Kaun et al.'s filtered images. The SAR filters result in reduction of contrast along with smoothing of the images. Smoothing is also observed on usage of AD, NSS, TV, and ATV filters. The denoised images appear noisy on using filters such as wavelet shrinkage, TMED, ATMED, and GLM filters. The filters such as DsFls, DsFlsminsc, DsFca, HATMAV, FBF, FIF, and AD result in some amount of blurring of the images with loss of the texture information.

*Clinical Validation*. The clinical validation of preprocessed images is carried out by seeking opinion of four practicing cardiologists at PGI, Chandigarh. One of the authors of this paper is a senior practicing cardiologist, who initially graded the quality of the processed images. The grading of images is based on visual perception of the practicing clinicians. The procedure of grading consisted of awarding a grade value in the range of 1 to 10 where 1 signifies the poor quality of processed image and grade 10 is assigned to the best visual quality image. The analyses of these grades reveal that the filters such as the DsFgf4d, DsFhomog, DsFlsmv, DsFhmedian, DPAD, GLM, PSBE, NLM, and PPB are acceptable whereas DsFhomo, DsFmedian, DsFad, TMED, HTMED, and RNLA filtered images are clinically not suitable.

## 5. Conclusion

The despeckling applications of eleven types of filters are analyzed in this paper in terms of blind and full-reference parameters. The traditional parameters often fail to reflect the true performance of the filters in the absence of a noise-free reference image. The parameters such as speckle suppression index, speckle suppression and mean preservation index, and beta metric are used to study the speckle suppression and edge preservation capability of each filter. The image quality and structural preservation are analyzed using SMPI, FoM, IQI, and SSI. Various types of benchmark filters are available and it would be very difficult to choose the best clinically acceptable filter. It is also necessary to remove speckle noise but with the edges preserved. The major contributions of this paper are as follows: it helps in selecting the best filters suitable for clinical TTE images among eleven types of filters and their constituents, it studies and evaluates the performance in terms of blind and full-reference based parameters, and evaluations of filters based image quality metrics are validated by practicing clinicians. Based on the quantitative evaluation and clinical validation, it is concluded that the performances of DsFgf4d, DsFhomog, DsFlsmv, DsFhmedian, DPAD, GLM, PSBE, NLM, and PPB filters are acceptable.

## Figures and Tables

**Table 1:** Overview of despeckling methods and quality metrics computed by various researchers.

Reference	Types of filters	Number of filters	Performance parameters	Type of image	*β*/SMPI/SSI?
[1]	WLT, SAR, AD, GEO	15	FoM, SSIM, MSE, CNR, SNR	Heart US	No
[4]	LA, AD, MR, NLM, HYB	11	PSNR, MSE, SSIM, FoM	Breast US	No
[5]	DsF filters based on LS, WNR, MED, AD, GEO, HYBMED	10	MSE, SNR, PSNR, RMSE, QI, SSIM, AD, SC, NCC, MD, LMSE, NAE, Err	Carotid artery US	No
[3]	MED, AD, WLT, WNR, AVG, HYB	7	PSNR	Bone fracture	No
[10]	WLT, AMED, AD, MAP, FF, LLS	7	SNR, ENL, CNR, EKI, CPU time	OCT Retina	No
[21]	WLT, FF, NLM, TV, RNLA, HFF	17	PSNR, MSE, SNR, FoM, CoC, *β*, SSIM	Heart US	*β*
[12]	AMED, WNR, LS, MBR, AD, BS	17	PSNR, SNR, SSIM, FoM, EKI, MVR	Prostate US	No
[6]	MED, AMED, FIF, FBF, WLT, HFIF	10	*β*, MSE, SNR, PSNR	Kidney US	*β*
[7]	MED, Le-Sig, LR, Lee, Frost, MAP	07	SSI, EEI, FPI, IDPC	SAR image	*β*, SSI
[8]	Lee, Frost, MAP, WLT, BM3D, PPB	07	ENL, SSI, SMPI, CoC, ESI	SAR image	Yes
This paper	WLT, MR, FF, NLM, DsF, AD, BLT, TV, GEO, SAR, FIF (eleven types)	45	18 parameters along with visual quality assessment and clinical validation	TTE images	Yes

WLT: wavelet; LA: local adaptive; US: ultrasound; GEO: geometric; HYB: hybrid; MED: median; HYBMED: hybrid median; WNR: Wiener; LS: local statistics; MR: multiresolution; AMED: adaptive median; FF: fuzzy filters; HFF: hybrid fuzzy filters; LLS: linear least square; FIF: Fourier ideal filter; FBF: Fourier Butterworth filter; HFIF: homomorphic FIF; LR: local region; Le-Sig: Lee-Sigma; MAP: maximum a posteriori; DsF: despeckling filter; BLT: bilateral; FoM: figure of merit; CoC: correlation coefficient; SC: structural content; LMSE: Laplacian mean square error; MD: maximum difference; Err3 and Err4: normalized error summation; NAE: normalized average error; NCC: normalized cross-correlation; OCT: optical computed tomography; SAR: synthetic aperture radar; AVG: average; RNLA: Ripplet nonlinear approximation; MBR: M-band ridgelet; BS: BlockShrink; PPB: probabilistic patch based; MAP: maximum a priori.

**Table 2:** Types of despeckling techniques.

*Anisotropic diffusion*: AD [1, 31], CED [1, 33], DPAD [4, 34], SRAD [1, 32]	*SAR*: Lee [1, 7, 8, 23], Kaun [1, 7, 8, 36], Frost [4, 37]	*Wavelet shrinkage*: BayesShrink [21, 25], OWT [21, 26], MPT [21, 27], ProbShrink [21, 30], soft thresholding [3, 6], NeighShrink [28], SURELET [21, 29]
*Total variation*: TV [12, 18], AFTV [16], ATV [17]	*Nonlocal means*: OBNLM [14], PPB [15]	

*Despeckling filters*: DsFlsminsc, DsFlsmv, DsFhomog, DsFwiener, DsFmedian, DsFhmedian [5, 9, 12]	*Fuzzy*: TMED, TMAV, ATMED, TMAV, HTMAV, HTMED, HATMED, GWF [21, 24]	*Multiscale techniques*: GLM [1, 4, 13, 21], MBR [9, 38], RNLA [11, 12], PSBE [10]

Fast bilateral filter [12, 20]	Geometric filter [1, 5, 35]	*Fourier*: FIF, FBF, HFIF, HFBF [6]

MPT: multiscale product thresholding; BayesShrink: Bayes thresholding; OWT: orthogonal wavelet thresholding; SURE: Stein's unbiased risk estimation; LET: linear expansion of threshold; NeighShrink: neighborhood shrinkage; PSBE: posterior sampling based Bayesian estimation; GLM: generalized likelihood ratio filtering method; TV: total variation.

**Table 3:** Input parameters of despeckling techniques.

Method(s)	Parameters
DsFlsminsi and DsFlsmv [5, 22]	Window size = 5 × 5; iterations = 2, also with 3 × 3, 7 × 7, and 9 × 9
FBL [20]	Width of spatial Gaussian = 10; width of range Gaussian = 20
Fuzzy filter [21, 24]	Window size = 3 × 3; padding, also with 5 × 5, 7 × 7, and 9 × 9
FIF/HFIF and FBF/HFBF [21, 24]	*f*_*c*_ = 500, with order = 2, also with *f*_*c*_ = 100 and 1000
HDWT and HDTDWT [3]	Window size = 3 × 3; level = 2
DsFhomog [5, 22]	Window = 3 × 3
OBNLM [4, 14]	Search area = 23 × 23; block size = 15 × 15; smoothing value *h* = 0.4
Lee, Kaun, and Frost [1, 37]	Window size = 5 × 5
ProbShrink [30]	Window size = 3 × 3; level = 2; sym8
AFTV [16]	Iterations = 3; *λ* = 0; time step Δ*t* = 0.2
SURELET [29]	Downsampling *N* = 4; overlap factor *K* = 3; redundancy *K*_2_ = 3
RNLA [11, 12]	Support *c* = 1; degree *d* = 3
NSS [28]	Level = 3; sym8
MBR [9]	Band M = 3; *α* = 5000
ATV [17]	Iterations = 2; *λ* = 1; time step Δ*t* = 0.2
DsFad [1]	Diffusion constant = 30; rate of diffusion = 0.25; iterations = 20
DsFsrad [1, 5]	Iterations = 30; time step Δ*t* = 0.02; *ρ* = 1
CED [1]	Iterations = 20; time step Δ*t* = 0.02; diffusion constant = 20
DPAD [4, 34]	Iterations = 30; time step Δ*t* = 0.02; Cu noise estimation
DsFgf4d [5, 22, 35]	Window = 3 × 3; iterations = 2
DsFmedian [5, 22]	Window = 5 × 5; iterations = 3
PPB [4, 15]	Iterations = 4; *α* = 0.8; *T* = 2; search area = 23 × 23; patch size = 7 × 7
DsFhmedian [5, 22]	Window = 5 × 5; iterations = 2
Hybrid fuzzy [21]	Fuzzy and Wiener window size = 3 × 3
ROF [18]	Time step Δ*t* = 0.25; iterations = 5
PSBE [10]	Spatial sigma = 0.01; window size = 21 × 21; samples = 100
MPT [27]	Scale number = 2; *C* = 12
BayesShrink [25]	wtype = db4; level = 2
GLM [13]	Window size = 3 × 3; level = 2; *K* = 3
DsFwiener [5, 22]	Window size = 5 × 5, 3 × 3, and 7 × 7

Spatial sigma refers to spatial variance of the initial probability distribution; wtype refers to wavelet type.

**Table 4:** Comparison of performance metrics for wavelet shrinkage and multiresolution filters.

Methods	IQI	*β*	FoM	SMPI	SSI	Soft threshold	IQI	*β*	FoM	SMPI	SSI
MPT	**0.9332**	0.8601	**0.8937**	2.806	0.9868	RBIO4.4	0.3678	0.0084	0.7991	58.59	0.9719
RNLA	0.4156	0.8428	0.8122	2.976	0.9627	DB2	0.5527	0.0226	0.8271	58.95	0.9672
OWT	0.4045	0.4477	0.9147	3.070	1.0409	DB4	0.3999	0.0129	0.8045	58.74	0.9638
MBR	0.5793	0.2312	0.7853	2.538	0.9341	DB8	0.1583	0.0253	0.7561	59.91	0.9481
NSS	0.5668	0.4259	0.8223	**0.962**	**0.6904**	DB45	0.0054	0.0091	0.5825	54.38	**0.9152**
BSHRINK	0.4358	**0.9996**	**0.9995**	2.589	0.9999	COIF1	0.5036	0.0302	0.8359	59.17	0.9650
SURELET	0.4119	0.8958	**0.9692**	3.967	0.9887	COIF5	0.0420	0.0029	0.7161	58.71	0.9454
GLM	0.7277	**0.9866**	**0.8963**	**2.238**	0.9963	SYM2	0.5527	0.0226	0.8271	58.95	0.9672
PSBE	**0.9246**	**0.9805**	**0.9923**	**2.504**	0.9960	SYM8	0.1250	0.0177	0.7694	55.92	0.9750
PS (DB2)	0.5026	0.1939	0.8321	27.61	**0.7266**	DMEY	0.0158	0.0072	0.5832	52.73	0.9293
PS (DB4)	0.3623	0.1530	0.8366	20.55	0.7281	BIOR1.1	**0.6619**	0.2762	**0.8785**	59.52	0.9588
PS (DB8)	0.2244	0.1350	0.8368	17.44	0.7287	BIOR1.5	0.3929	0.0019	0.8033	58.54	0.9754
PS (SYM2)	0.4547	0.1939	0.8321	27.61	**0.7266**	BIOR6.8	0.1598	0.0136	0.7634	57.11	0.9692
PS (SYM4)	0.3479	0.1530	0.8366	20.55	0.7281	RBIO1.1	**0.6619**	0.2762	**0.8785**	59.52	0.9588
PS (SYM8)	0.2223	0.1350	0.8368	17.44	0.7287	RBIO2.2	0.5927	0.0359	0.8252	58.73	0.9581

**Table 5 tab5:** Comparison of performance parameters for DsF, Fourier, and SAR filters.

Methods	IQI	*β*	FoM	SMPI	SSI
DsFlsmv	**0.9433**	**0.9301**	**0.9628**	**2.364**	0.9810
DsFwiener	0.8580	**0.9744**	**0.9515**	**2.292**	0.9884
DsFmedian	**0.9403**	0.7116	**0.9277**	**2.922**	0.9899
DsFlsminsi	0.8179	0.1045	0.8553	2.960	0.9781
DsFls	0.7042	0.4583	0.7112	4.364	**0.8695**
DsFhomog	0.7352	0.6839	0.7305	5.849	1.0749
DsFhomo	0.8101	0.1698	0.7901	3.597	0.9886
DsFgf4d	0.8588	0.5519	0.8802	7.308	0.9645
DsFwaveltc	0.5576	**1.0000**	**1.0000**	2.588	1.0000
DsFsrad	0.7705	**0.9848**	**0.9272**	**2.663**	0.9955
DsFlecasort	0.8771	0.5258	0.8978	4.002	0.9941
DsFca	0.8372	0.5899	0.8247	3.154	0.9261
DsFad	0.5093	0.8111	0.8799	3.358	0.9608
FIF	0.4109	**0.9511**	**1.0000**	2.587	0.9994
FBF	0.4242	**0.9747**	**0.9955**	2.575	0.9934
HFIF	0.3904	0.8045	**0.9944**	3.733	0.9756
HFBF	0.3961	**0.9205**	**0.9682**	3.381	**0.9645**
CED	**0.9769**	0.8964	**0.9785**	**2.516**	0.9803
Lee	**0.9265**	**0.9906**	**0.9432**	6.201	1.1604
Frost	0.7923	0.6164	0.7615	6.019	1.1044
Kaun	**0.9241**	**0.9829**	**0.9432**	6.399	1.1704
DPAD	0.5824	**0.9837**	**0.9797**	**2.325**	0.9966
FBL	0.7712	**0.9714**	0.8079	**2.292**	0.9927
PPB	0.3951	**0.9409**	**0.9882**	2.931	**0.9701**
NLM	0.5869	0.8891	0.8034	**2.367**	0.9853
MED	0.8403	0.0460	0.8523	3.378	0.9884

**Table 6:** Comparison of performance parameters for total variation and fuzzy based filters.

Method	IQI	*β*	FoM	SMPI	SSI
AFTV	0.2443	0.6672	0.6343	**2.044**	0.9583
TV	**0.8488**	0.4390	**0.8348**	3.085	**0.9462**
ATV	0.2916	**0.9433**	**0.8338**	2.162	0.9766
TMED	0.7539	0.0666	0.8869	3.790	1.0001
ATMED	**0.8785**	0.4438	**0.9514**	2.881	0.9865
TMAV	0.8174	0.1681	**0.9145**	3.767	0.9999
HTMED	0.7616	0.0074	0.8620	4.015	0.9974
HATMED	0.8470	0.4467	**0.9171**	3.082	0.9834
HTMAV	0.8008	0.2127	0.8883	3.905	0.9978
GWF	0.8375	**0.9634**	**0.9407**	2.831	0.9804
GWF1	0.7462	0.1349	0.8608	2.804	**0.9675**
GWF2	0.8119	0.4145	**0.9035**	**2.234**	**0.9682**

**Table 7:** Comparison of performance parameters (average ± standard deviation).

Methods	IQI	*β*	FoM	SMPI	SSI	MD
DsFlsmv	**0.947 ± 0.003**	0.939 ± 0.006	0.962 ± 0.007	2.661 ± 0.260	0.974 ± 0.005	92.167 ± 6.873
DsFwiener	0.865 ± 0.005	0.973 ± 0.004	0.946 ± 0.016	2.573 ± 0.259	0.989 ± 0.001	**28.493 ± 2.104**
DsFmedian	**0.938 ± 0.004**	0.707 ± 0.009	0.939 ± 0.010	2.957 ± 0.292	0.984 ± 0.006	212.16 ± 4.13
DsFgf4d	0.867 ± 0.015	0.555 ± 0.011	0.903 ± 0.015	7.356 ± 0.436	0.962 ± 0.006	245.00 ± 10.00
DsFsrad	0.785 ± 0.032	**0.987 ± 0.003**	0.947 ± 0.011	2.682 ± 0.290	**0.994 ± 0.001**	**12.770 ± 0.319**
FIF	0.389 ± 0.048	0.950 ± 0.001	**1.000 ± 0.000**	2.628 ± 0.292	0.999 ± 0.000	42.002 ± 5.115
FBF	0.404 ± 0.048	0.974 ± 0.000	**0.996 ± 0.001**	2.597 ± 0.295	0.991 ± 0.002	64.09 ± 2.00
GLM	0.727 ± 0.019	**0.991 ± 0.006**	0.924 ± 0.032	2.571 ± 0.258	0.997 ± 0.002	**38.21 ± 0.02**
MPT	**0.950 ± 0.011**	0.868 ± 0.008	0.926 ± 0.020	2.790 ± 0.272	0.984 ± 0.002	162.00 ± 20.45
RNLA	0.410 ± 0.016	0.843 ± 0.014	0.810 ± 0.024	3.041 ± 0.276	**0.950 ± 0.008**	122.50 ± 11.46
MBR	0.599 ± 0.022	0.228 ± 0.009	0.802 ± 0.021	**2.498 ± 0.240**	0.907 ± 0.020	214.97 ± 12.2
ATV	0.281 ± 0.025	0.947 ± 0.005	0.841 ± 0.035	**2.447 ± 0.253**	0.969 ± 0.005	45.698 ± 0.514
PPB	0.379 ± 0.045	0.947 ± 0.004	**0.984 ± 0.012**	3.279 ± 0.263	0.963 ± 0.007	96.639 ± 0.400
ATMED	0.876 ± 0.005	0.443 ± 0.010	0.948 ± 0.011	3.165 ± 0.264	0.978 ± 0.009	202.93 ± 10.12
HATMED	0.845 ± 0.008	0.449 ± 0.007	0.917 ± 0.016	3.354 ± 0.265	0.972 ± 0.011	212.29 ± 0.07
GW filter	0.837 ± 0.007	0.968 ± 0.005	0.921 ± 0.025	3.137 ± 0.284	0.974 ± 0.003	**30.80 ± 1.68**
GWF2	0.810 ± 0.010	0.418 ± 0.009	0.898 ± 0.020	2.549 ± 0.265	**0.954 ± 0.010**	207.98 ± 0.06
DPAD	0.577 ± 0.026	**0.989 ± 0.003**	**0.990 ± 0.005**	2.639 ± 0.264	0.997 ± 0.000	**29.825 ± 4.202**
PSBE	**0.923 ± 0.002**	**0.980 ± 0.001**	**0.991 ± 0.002**	2.817 ± 0.269	0.993 ± 0.003	89.276 ± 6.414
FBL	0.766 ± 0.019	0.973 ± 0.002	0.818 ± 0.039	2.638 ± 0.260	0.992 ± 0.001	51.058 ± 14.409

**Table 8:** Comparison of traditional performance parameters (average ± standard deviation) (the values are computed using 1000 TTE images).

Methods	MSE	RMSE	Err3	Err4	MSSIM	NCC	LMSE	NAE	SNR (dB)	PSNR (dB)
DsFlsmv	23.73 ± 1.81	**4.86 ± 0.18**	9.09 ± 0.23	13.44 ± 0.38	**0.994 ± 0.001**	0.965 ± 0.007	0.206 ± 0.015	**0.070 ± 0.004**	39.91 ± 1.39	34.39 ± 0.34
DsFwiener	**6.46 ± 1.01**	**2.55 ± 0.19**	**3.86 ± 0.21**	5.04 ± 0.21	**0.994 ± 0.001**	**0.990 ± 0.001**	**0.055 ± 0.008**	**0.049 ± 0.002**	**51.04 ± 0.68**	**40.07 ± 0.68**
DsFmedian	60.51 ± 2.41	7.77 ± 0.15	18.26 ± 0.24	29.88 ± 0.34	0.978 ± 0.002	0.950 ± 0.013	0.500 ± 0.013	**0.064 ± 0.007**	31.53 ± 2.24	30.32 ± 0.17
DsFgf4d	267.5 ± 18.9	16.34 ± 0.57	33.47 ± 0.78	50.94 ± 0.83	0.927 ± 0.007	**1.005 ± 0.006**	0.801 ± 0.015	0.171 ± 0.017	18.63 ± 2.07	23.86 ± 0.31
DsFsrad	**2.65 ± 0.62**	**1.61 ± 0.19**	**2.36 ± 0.21**	**2.98 ± 0.21**	**0.996 ± 0.001**	**0.998 ± 0.001**	**0.027 ± 0.006**	**0.034 ± 0.002**	**58.93 ± 0.96**	**44.00 ± 1.03**
FIF	**3.21 ± 0.12**	**1.79 ± 0.04**	**3.44 ± 0.04**	5.26 ± 0.08	**1.00 ± 0.00**	**0.999 ± 0.000**	**0.098 ± 0.002**	**0.035 ± 0.006**	**57.05 ± 2.18**	**43.07 ± 0.21**
FBF	**4.87 ± 0.22**	**2.20 ± 0.04**	**4.56 ± 0.05**	7.04 ± 0.04	**1.00 ± 0.00**	**0.991 ± 0.002**	**0.088 ± 0.001**	**0.027 ± 0.003**	**53.44 ± 2.19**	**41.27 ± 0.17**
GLM	**2.52 ± 1.78**	**1.48 ± 0.57**	**2.28 ± 0.73**	**3.24 ± 0.65**	**0.992 ± 0.006**	**0.997 ± 0.002**	**0.018 ± 0.011**	**0.030 ± 0.012**	**61.86 ± 7.56**	**45.37 ± 3.77**
MPT	32.14 ± 4.36	5.65 ± 0.38	10.84 ± 0.25	16.84 ± 0.17	0.986 ± 0.006	0.966 ± 0.004	0.248 ± 0.015	**0.076 ± 0.002**	37.12 ± 1.24	33.11 ± 0.59
RNLA	43.82 ± 7.31	6.59 ± 0.54	9.89 ± 0.67	13.48 ± 0.75	0.890 ± 0.012	0.975 ± 0.004	0.292 ± 0.024	0.156 ± 0.014	34.43 ± 1.44	31.76 ± 0.71
MBR	179.84 ± 8.52	13.40 ± 0.31	24.69 ± 0.31	36.13 ± 0.34	0.924 ± 0.008	0.891 ± 0.026	0.964 ± 0.001	0.209 ± 0.026	22.07 ± 2.12	25.58 ± 0.21
ATV	19.73 ± 2.49	**4.43 ± 0.27**	6.98 ± 0.25	9.35 ± 0.17	0.964 ± 0.008	0.971 ± 0.005	0.128 ± 0.010	**0.086 ± 0.006**	41.54 ± 1.19	35.21 ± 0.56
PPB	**6.54 ± 0.44**	**2.55 ± 0.08**	5.31 ± 0.06	9.04 ± 0.09	0.928 ± 0.007	**1.003 ± 0.000**	0.106 ± 0.008	**0.060 ± 0.008**	**51.09 ± 1.46**	39.98 ± 0.29
ATMED	103.95 ± 3.42	10.19 ± 0.16	22.65 ± 0.26	35.48 ± 0.25	0.973 ± 0.002	0.920 ± 0.018	0.804 ± 0.009	**0.098 ± 0.010**	27.05 ± 2.02	27.96 ± 0.14
HATMED	123.06 ± 4.92	11.09 ± 0.22	23.59 ± 0.29	36.38 ± 0.33	0.961 ± 0.003	0.899 ± 0.022	0.804 ± 0.005	0.123 ± 0.012	25.59 ± 1.96	27.23 ± 0.17
GW filter	10.14 ± 1.44	**3.17 ± 0.23**	**4.69 ± 0.23**	5.86 ± 0.23	0.988 ± 0.002	**1.001 ± 0.001**	**0.068 ± 0.009**	**0.066 ± 0.004**	47.34 ± 0.95	38.11 ± 0.63
GWF2	102.35 ± 3.36	10.15 ± 0.16	21.78 ± 0.21	34.04 ± 0.24	0.967 ± 0.003	0.935 ± 0.017	0.826 ± 0.008	0.121 ± 0.011	27.18 ± 1.94	28.03 ± 0.14
DPAD	**2.33 ± 0.93**	**1.49 ± 0.31**	**2.82 ± 0.46**	**4.17 ± 0.58**	**0.999 ± 0.001**	**1.000 ± 0.000**	**0.022 ± 0.007**	**0.018 ± 0.002**	**60.72 ± 1.83**	**44.82 ± 1.87**
PSBE	**8.31 ± 0.36**	**2.83 ± 0.06**	6.63 ± 0.09	10.65 ± 0.13	**0.998 ± 0.000**	0.977 ± 0.005	**0.060 ± 0.001**	**0.025 ± 0.003**	48.99 ± 2.09	38.97 ± 0.19
FBL	11.64 ± 1.95	**3.41 ± 0.28**	5.09 ± 0.26	6.55 ± 0.18	0.971 ± 0.008	0.985 ± 0.003	**0.055 ± 0.004**	**0.071 ± 0.005**	46.17 ± 0.93	37.52 ± 0.78
